# Mapping of the Motor Cortex

**DOI:** 10.7759/cureus.10645

**Published:** 2020-09-25

**Authors:** Faisal R Jahangiri, Aksharkumar Dobariya, Aaron Kruse, Olga Kalyta, John D Moorman

**Affiliations:** 1 Neurophysiology, Axis Neuromonitoring, Richardson, USA; 2 School of Behavioral and Brain Sciences, The University of Texas at Dallas, Richardson, USA; 3 Neurophysiology, Global Innervation, LLC, Dallas, USA; 4 Neurology and Neurotherapeutics, The University of Texas Southwestern Medical Center, Dallas, USA

**Keywords:** brain tumor, neuro-surgery, motor mapping, cortical mapping, ionm, eeg, neuro-monitoring, electrocorticography

## Abstract

The resection of brain tumors located within or near the eloquent tissue has a higher risk of postoperative neurological deficits. The primary concerns include loss of sensory and motor functions in the contralateral face, upper and lower extremities, as well as speech deficits. Intraoperative neurophysiological monitoring (IONM) techniques are performed routinely for the identification and preservation of the functional integrity of the eloquent brain areas during neurosurgical procedures. The IONM modalities involve sensory, motor, and language mapping, which helps in the identification of the boundaries of these areas during surgical resection. Cortical motor Mapping (CmM) technique is considered as a gold-standard technique for mapping of the brain. We present the intraoperative CmM technique, including anesthesia recommendations, types of electrodes, as well as stimulation and recording parameters for successful monitoring.

## Introduction

The first documented cortical tumor resection was performed by two neurologists A. Hughes Bennett and Rickman J. Godlee in 1884, London [1]. It resulted in the patient passing away 28 days afterward due to complications from the procedure. There are a variety of ways in which one can classify the different kinds of tumors. One such broad division can be made based on tumor malignancy characteristics; if the tumor is prone to encroaching on other areas of the body (i.e., malignant) or if it is non-cancerous (i.e., benign). Another method categorizes a tumor as per its origin, which can be termed as primary (i.e., originating in the brain or spinal cord) or secondary (i.e., originating from somewhere else in the body). With primary brain tumors, we can further group them based on the type of cell causing the tumor mass. A glioma is a tumor that originates from glial cells and is known to be the most common form of malignant primary brain tumor. Some other kinds of primary tumors include meningiomas, ependymomas, and many types of lymphomas.

The patients who have undergone tumor resection surgery can suffer from deficits that can reduce their quality of life. Some of the common symptoms caused by motor cortex tumors include hemiparesis or hemiplegia, myopathy, ataxia, gait dysfunction, and spasticity. These symptoms justify the need for surgery to alleviate deficits and improve the quality of life for patients if alternative therapeutic effects have failed. Furthermore, it also demonstrates the need for a safe resection where the odds of patients developing postoperative deficits are reduced. The intraoperative neurophysiological monitoring (IONM) of changes to the nervous system caused by surgical manipulations not only helps with lowering postoperative deficits, but it also acts as an alarm system to warn and guide the surgeon. Cortical tumors surrounding the primary motor and premotor areas are likely to cause the symptoms above.

There are two approaches used in tumor resection, gross-total resection (GTR) and subtotal resection (STR). GTR involves the total removal of the tumor. It can be challenging to perform in cortical motor surgeries due to the necessity to differentiate between abnormal and healthy tissue. In contrast, STR calls for the removal of only the necessary parts of the tumor that can potentially alleviate the motor symptoms. Operating on motor regions of the brain becomes even more treacherous when considering how tumor masses may have distorted the anatomical landmarks that surgeons employ for resection surgeries. Thus, it is extremely crucial to use methods of locating the functional anatomical regions such as the central sulcus, pre-central gyrus, post-central gyrus, and other functional brain region surrounding the tumor. Knowledge about these alterations caused by tumors allows for safer approaches to surgical resections and guards the patients against potential damage to motor areas as protective actions would be taken.

IONM techniques can help identify and find the correct path of the central sulcus (CS) across both hemispheres, while also mapping the cortical homunculus representation at the primary motor area. The method utilized to obtain such beneficial anatomical and functional knowledge during surgery is called motor mapping. Research had reported significant beneficial effects from motor mapping, such as the postoperative complication rate dropping from 21% to 13% when adequate neuro-monitoring was employed [2].

Fritsch and Hitzig (1870) pioneered the discovery of direct electrical cortical stimulation (DCS/DECS) of the animal brain [3]. Penfield and Boldrey demonstrated the mapping of motor, sensory, and language cortices by directly stimulating an open cortex in human patients [4]. Different scientists have made tremendous progress throughout the 19th and 20th centuries to improve cortical motor mapping. George Ojemann introduced the Ojemann Cortical Stimulator (OCS), an electrical stimulator that improved Penfield's technique of motor mapping. Taniguchi et al. proposed short multipulse stimulation using high frequencies during surgeries performed under general anesthesia [5]. In this technical report, we will discuss Penfield and Taniguchi's methods of motor mapping with other consideration such as required tools, anesthetic recommendations, and modalities with their parametric values.

## Technical report

Pre-operative evaluation of patient

The physiological signals of the human nervous system are unique to each individual, as the rest of the body characteristics directly influences them. Unlike some static biological phenomena such as cardiac rhythms, brain physiology is highly dynamic, and it can change drastically during surgical procedures due to manipulations and surgical anesthesia. Hence, it is essential to establish the baseline for modalities such as somatosensory evoked potentials (SSEP) and motor evoked potentials (MEP). Cortical and sub-cortical motor mapping is needed for procedures including but not limited to brain tumors, intracranial aneurysms, arteriovenous malformation (AVM), and epilepsy surgeries. A multimodality approach is needed for the best postoperative outcome, including cortical sensory mapping with phase reversal, cortical motor mapping, subcortical motor mapping, electromyography (EMG), and electrocorticography (ECoG).

A detailed patient medical and surgical history should be taken and documented with any previous factors that may affect intraoperative cortical mapping data.

Anesthesia

The patient can be placed under general anesthesia for procedures that do not require an evaluation of the voluntary motor and language functions during surgery. When a patient's motor functions require multiple assessments or when language mapping is performed, awake craniotomy with the asleep-awake-asleep method of anesthesia must be employed. Hence, the patient will be placed under light anesthesia during the opening of the dura. After that patients will be awakened for functional assessments during surgery. Baseline recordings will be useful to account for the effect of anesthesia before surgical manipulation. Recommended anesthetic agents include a combination of propofol and an analgesic agent administered by the total intravenous anesthesia (TIVA) method. Other anesthetics that can be used include ketamine, etomidate, and benzodiazepines [6]. Dexmedetomidine, an alpha-adrenergic agonist, should be avoided due to its inhibitory effects on MEP [7]. Initially, inhalation anesthetics may be used for the intubation of the patient, as significant TIVA transition will not affect recordings after incision [8]. However, inhalation anesthetics used for the entirety of the procedure can contribute to more postoperative deficits for patients, as thresholds for MEPs are higher in patients under inhalation anesthesia, leading to signals being weaker and more challenging to interpret. Also, it is important to note that the stability of the patient's temperature is critical to consider at the site of recording as low temperatures increase signal latencies [6].

Intraoperative neurophysiological monitoring (IONM)

**Figure 1:**
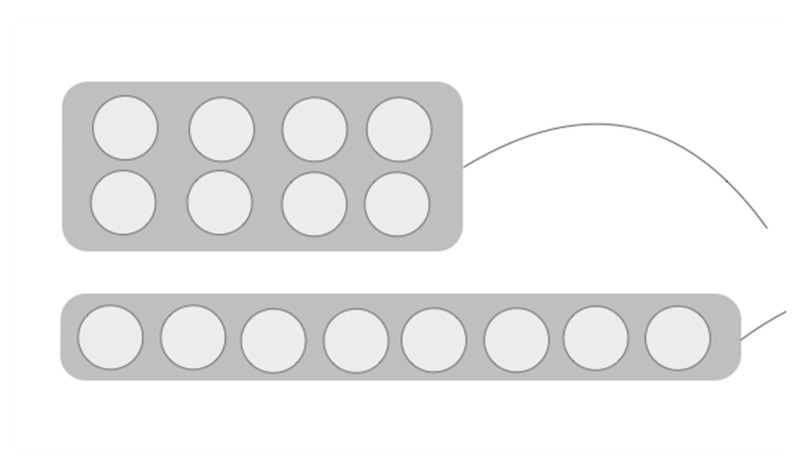
Subdural grids. Schematic presentation of the subdural cortical grids. A: Eight-contact grid (2 x 4); B: Eight-contact grid (1 x 8).

After the patient positioning on the operating table under anesthesia, subdermal needle electrodes are placed over the scalp as per the international 10-20 system for recording SSEP and EEG. Subdermal needle electrodes are placed in the face, upper and lower extremities muscles contralateral to the surgical site for recording EMG and MEP (Table 1). Surface adhesive electrodes are placed for stimulating peripheral (median, ulnar, posterior tibial, or femoral) nerves for SSEP and sensory mapping [8]. A phase reversal with sensory mapping is performed by placing a subdural grid or strip of the exposed cerebral cortex and stimulating the contralateral peripheral nerves. These grid electrodes are made of either stainless steel or platinum embedded in flexible silicone. Before performing the motor mapping, it is essential to localize the central sulcus (CS) correctly and to identify a potential shift in the sensory or motor cortices due to the physical expansion of the tumor mass. A 2 x 4 grid electrode is preferred for locating the CS, although other configurations are used as well, such as a 1 x 6 or 1 x 8 electrode strips. Cortical grids or strip can be used for cortical stimulation in addition to the handheld monopolar or bipolar probes (Figure 1).

**Table 1:** Electromyography. Recommended muscle recordings for electromyography (EMG) and direct electrical cortical stimulation (DECS) and subcortical stimulation.

Facial Muscles	Upper Extremities	Lower Extremities
Orbicularis Oculi	Deltoid	Quadriceps
Orbicularis Oris	Biceps Brachii	Tibialis Anterior
Tongue muscle	Flexor Carpi Ulnaris	Gastrocnemius
	Brachioradialis	Abductor Hallucis
	Abductor Pollicis Brevis	Extensor Hallucis Brevis
	Abductor Digiti Minimi	
	First Dorsal Interosseous	

Two techniques, Penfield and Taniguchi, have evolved for intraoperative cortical and subcortical mapping of the corticospinal tracts. Either of these two methods can be utilized based on tumor location, patient history, surgical procedure, and other factors.

A) Penfield cortical (50-Hz) technique

**Figure 2:**
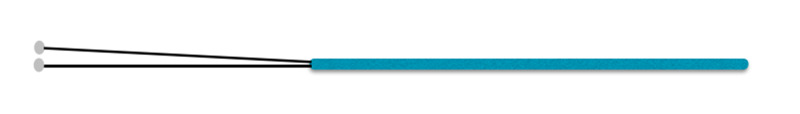
Bipolar probe. Schematic presentation of a bipolar ball tip handheld probe.

A distinguished neurosurgeon, Dr. Wilder Penfield, first described this technique in 1937. This technique is accomplished with a handheld bipolar stimulator using a 50-Hz stimulation (interstimulus interval of 20 milliseconds) with a train of monophasic cathodal pulses of an individual pulse width of 0.5 milliseconds [4]. The stimulation is applied to the cortex for a duration of 2 to 5 seconds, with a 5-10 seconds interval between each stimulus (Figure 2). Each stimulation point should not be stimulated consecutively. Direct cortical stimulation is performed at a lower intensity to the exposed cortex, starting from 2.0 milliamperes (mA). The stimulus should be increased in increments until either a positive response, maximum allowable stimulation of 20 mA is reached, or if after discharges (AD) are seen in ECoG recordings. If ADs are present, iced cold saline solution (4˚C) should be quickly applied to the exposed stimulated cortex.

Cortical areas of the face, tongue, arms, hands, legs, and feet are identified with stimulation and muscle recording. A subdural grid can also be placed over the area to monitor the status of the patient's motor function and to alert the surgeon of any changes that might later incur deficits for the patient [4]. Recording and stimulation parameters suggested for the Penfield method are specified in Table 2 and Table 3, respectively.

**Table 2:** Recording parameters. Recording parameters suggested by Penfield and Taniguchi. Hz = Hertz, µs = microseconds, µV = microvolts, div = division, ms = milliseconds, kΩ = kiloohms.

Recording Parameters		
Specification	Penfield	Taniguchi
Low-cut filter	10 Hz	10 Hz
High-cut filter	5000 Hz	5000 Hz
Notch Filter`	Off	Off
Dynamic Range (Input Gain)	200-500 µV/div	200-500 µV/div
Sensitivity	200 µV	200 µV
Time-base	100 ms/div	10 ms/div
Electrode impedance	> 5 kΩ	> 5 kΩ

**Table 3:** Stimulation parameters. Recommended stimulation parameters for Penfield and Taniguchi. Hz = Hertz, µs = microseconds, mA = milliamperes, s = seconds, ms = milliseconds.

Stimulation Parameters		
Specification	Penfield	Taniguchi
Type of Stimulator	Bipolar	Monopolar
Type of Pulse (Phase)	Biphasic or Monophasic	Monophasic Anodal
Frequency	50 Hz	250-500 Hz
Pulse width	300 - 1000 µs	500 µs
Intensity	2 - 20 mA	2 - 20 mA
Duration of stimulation	2 - 5 s	20 µs

B) Taniguchi cortical technique

**Figure 3:**
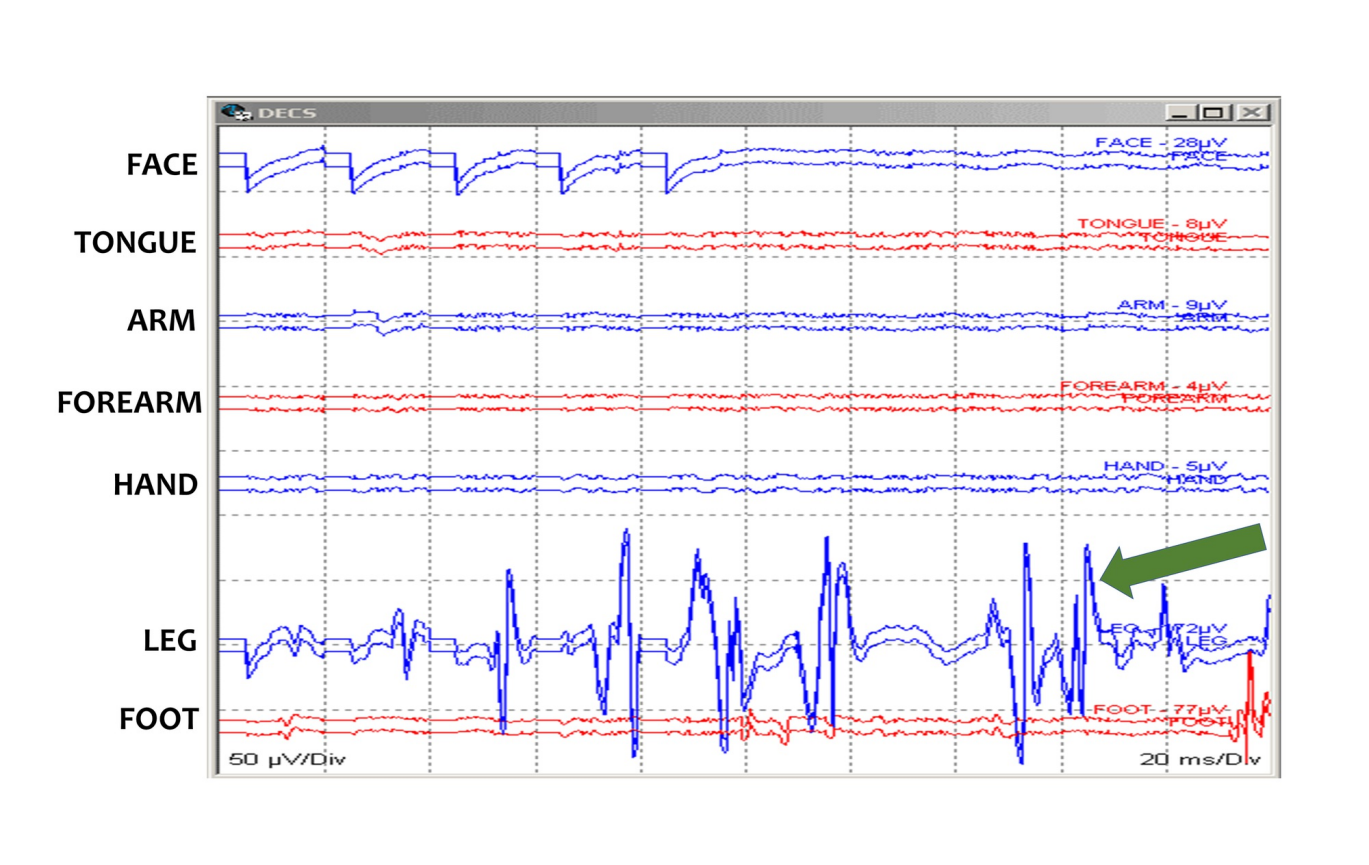
Motor mapping: Penfield method. Motor mapping responses after bipolar handheld stimulation using a Penfield 50 Hz method. Multiple responses are present in leg muscles (green arrow). Face (Orbicularis Oris), Tongue, Arm (Deltoid/Biceps Brachii), Forearm (Brachioradialis/Flexor Carpi Ulnaris), Hand (Abductor Pollicis Brevis/Abductor digiti minimi), Leg (Tibialis Anterior), and Foot (Abductor Hallucis) muscles.

**Figure 4:**
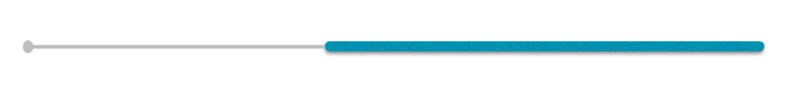
Monopolar probe. Schematic presentation of a handheld ball tip monopolar probe.

**Figure 5:**
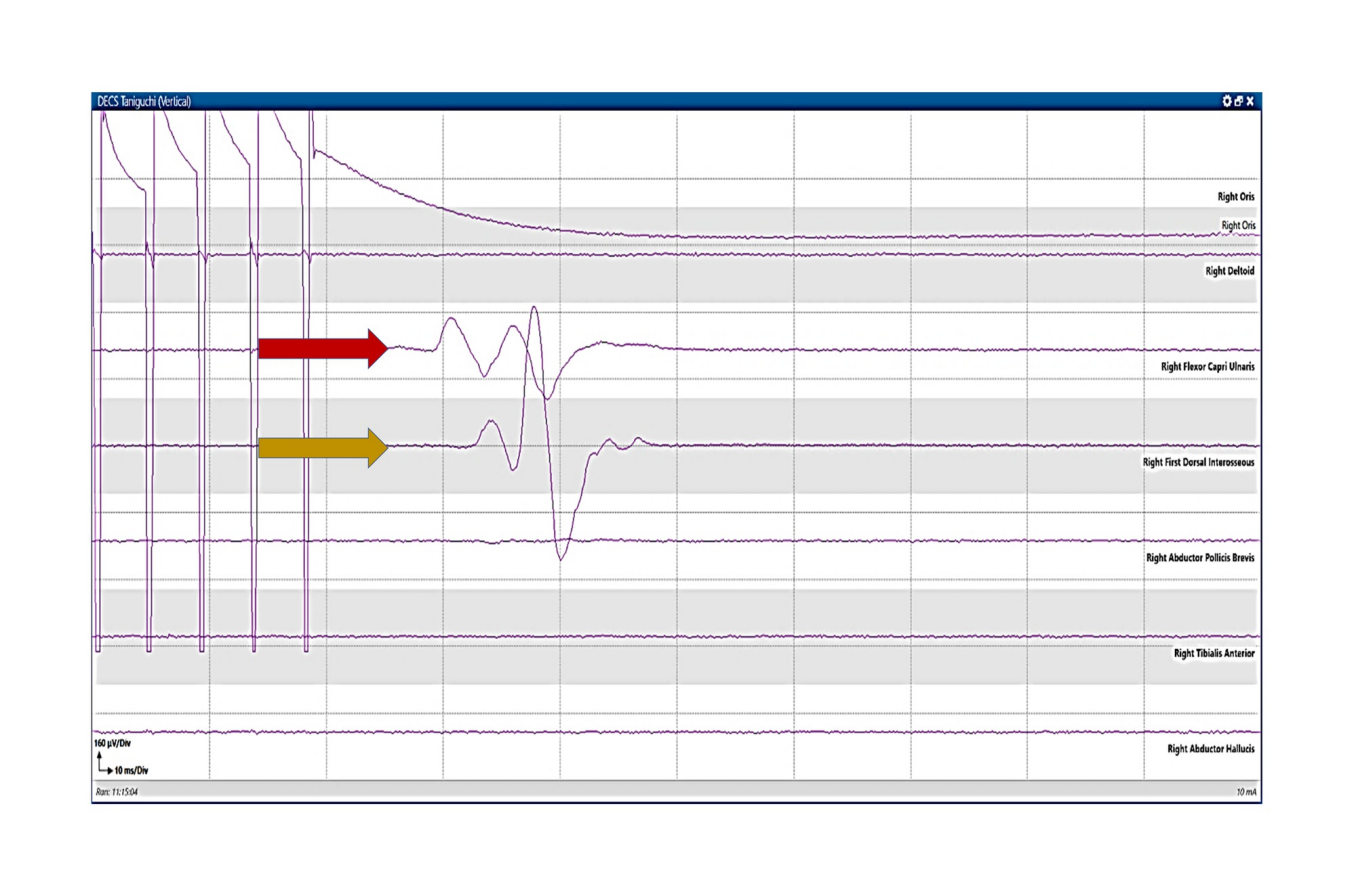
Motor mapping: Taniguchi method. Motor mapping responses after monopolar handheld stimulation using a Taniguchi high-frequency method. Motor evoked responses are present in the right Flexor Carpi Ulnaris (red arrow) and First Dorsal Interosseous (orange arrow) muscles.

**Figure 6:**
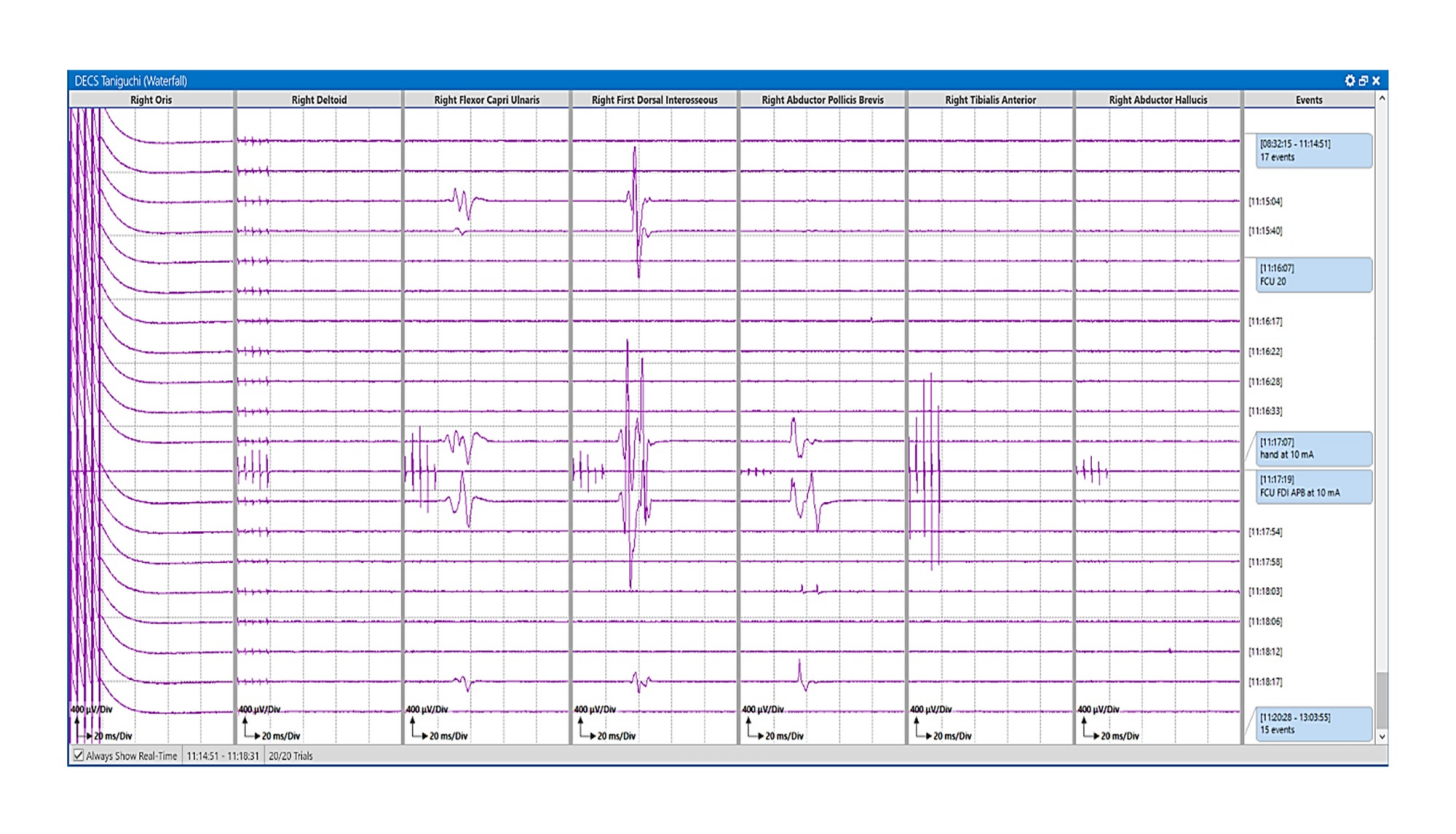
Motor mapping stack: Taniguchi method. Motor mapping responses in stack view after monopolar handheld stimulation using a Taniguchi high-frequency method. Motor evoked responses are stacked in the right Flexor Carpi Ulnaris, First Dorsal Interosseous, and Abductor Pollicis Brevis muscles.

In 1993, Taniguchi first published a high-frequency multipulse short train technique for direct cortical motor mapping. A handheld monopolar ball tip stimulator can be used to stimulate the motor cortex during the surgery to determine the status of the motor fibers at risk (Figure 3). This method uses a higher frequency of stimulation to the motor cortex at a rate of 250 to 500 Hz [4]. Electromyography (EMG) is used to record myogenic responses from the contralateral target muscles. This method provides the ability to monitor the functional integrity of corticospinal tracts throughout the procedure and alert the surgeon for any potential damage to the functional brain areas. Thus, it can help determine if further tumor resection can be continued or not (Figures 4-6). Recording and stimulation parameters suggested for the Taniguchi method are specified in Table 2 and Table 3, respectively.

Sub-cortical mapping

Sub-cortical motor mapping is used for tumors that are near or within the corticospinal tract (CST). A monopolar ball tip electrode is used in conjunction with the suction device used for tumor resection [9]. The subcortical CST fibers are identified by stimulation and recording the motor thresholds (MT), which act as an indicator of how far away from the CST the resection has taken place. MTs are measured in milliamps (mA), and every 1 mA change reflects a 1-mm distance change from the CST [9, 10]. The surgeon will continue to proceed until an MT of 7 mA is seen. Once MT of 7 mA is identified, it is generally recommended that the surgeon stops the resection of the tumor. Proceeding past 7 mA will stimulate the CST and will produce MEPs, and resection of tissue past this point increases the likelihood of having postoperative motor deficits in the patient [10]. Szelényi et al. showed that monopolar cathodal stimulation is more effective than the bipolar cathodal stimulus for eliciting MEPs for sub-cortical mapping (Table 4) [11].

**Table 4:** Sub-cortical stimulation. Sub-cortical mapping stimulation parameters. mA = milliamperes, ms = milliseconds, Hz = Hertz.

Sub-cortical Stimulation Parameters	
Intensity	2 - 20 mA
Type of stimulation	Monopolar Cathodal
Duration of pulse	0.5 - 1 ms
Frequency	250 - 500 Hz
Number of Pulses	4 - 5
Interstimulus Interval	3 - 4 ms

Electromyography (EMG)

Spontaneous electromyography (sEMG) signals from the contralateral face, upper and lower extremity muscles should be continuously recorded and monitored during the surgery [8]. EMG provides real-time feedback about any changes in muscle activity (Tables 1, 2).

Electroencephalography (EEG)

Electroencephalography (EEG) is a spontaneous recording of the electrical activity of the cerebral cortex recorded from the scalp. Scalp EEG consists of the summation of excitatory and inhibitory postsynaptic potentials of cortical pyramidal neurons. EEG is utilized to monitor the brain perfusion as well as the depth of anesthesia [8]. If burst suppression is noted it needs to be addressed immediately to provide an accurate neuromonitoring. It is important to maintain a consistent depth of anesthesia using spontaneous and processed EEG. This will allow us to avoid any variability in stimulation thresholds and accurate intraoperative cortical and subcortical stimulation (Table 5).

**Table 5:** Electroencephalography. Electroencephalography (EEG) recording parameters. Hz = Hertz, ms = milliseconds, µV = microvolts, div = division, kΩ = kiloohms.

EEG Recording Parameters	
Parameter	Value
Low-cut filter	1 Hz
High-cut filter	70 Hz
Notch filter	50 Hz (Europe/Asia) or 60 Hz (USA)
Dynamic Range (Input Gain)	30 µV/div
Sensitivity	70 µV/div
Sweep	1000 ms/div
Electrode impedance	> 5 kΩ

Electrocorticography (ECoG)

**Figure 7:**
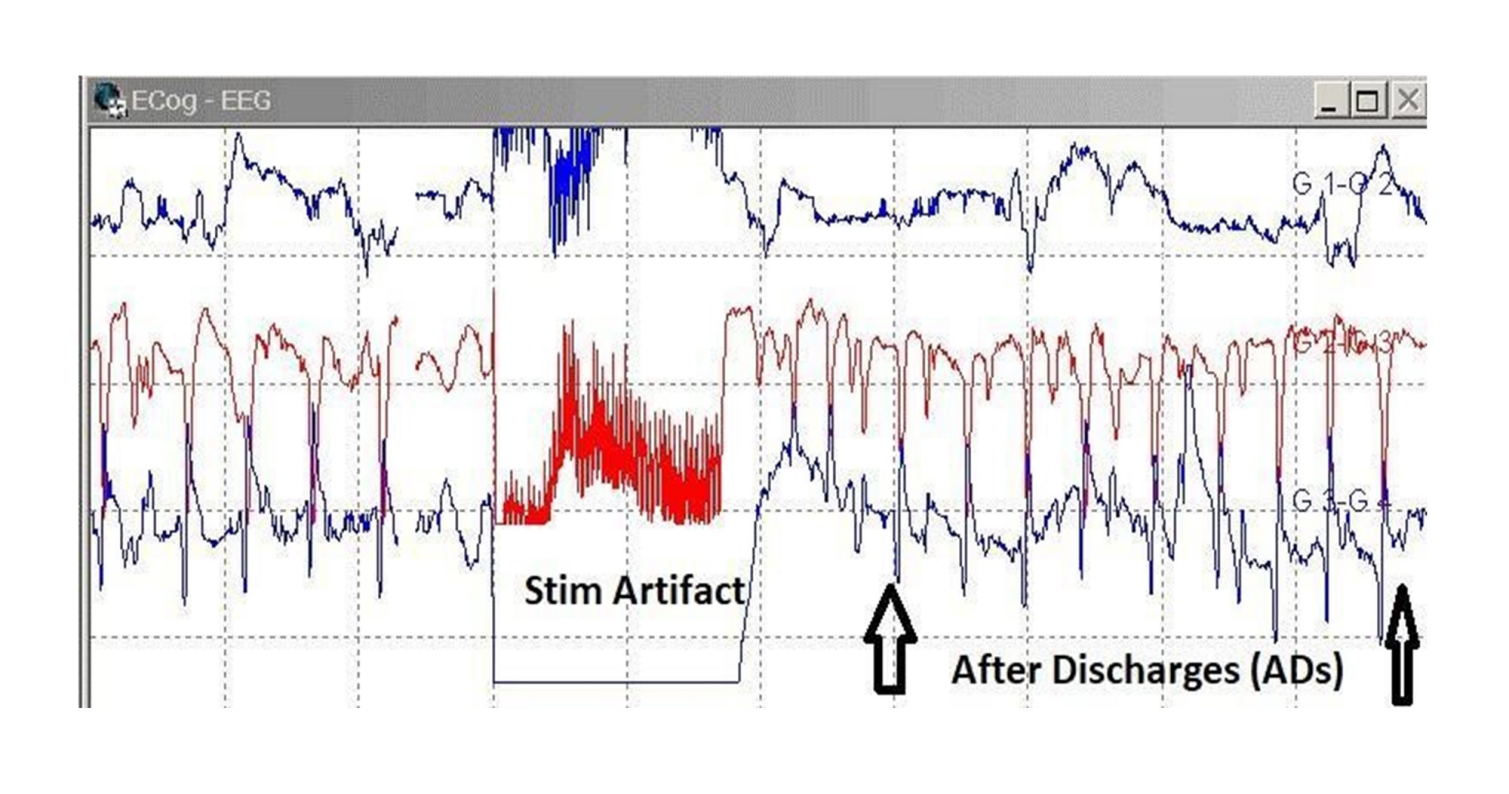
Electrocorticography. Electrocorticography (ECoG) recordings showing stimulation artifact induced after discharges (white arrows).

**Figure 8:**
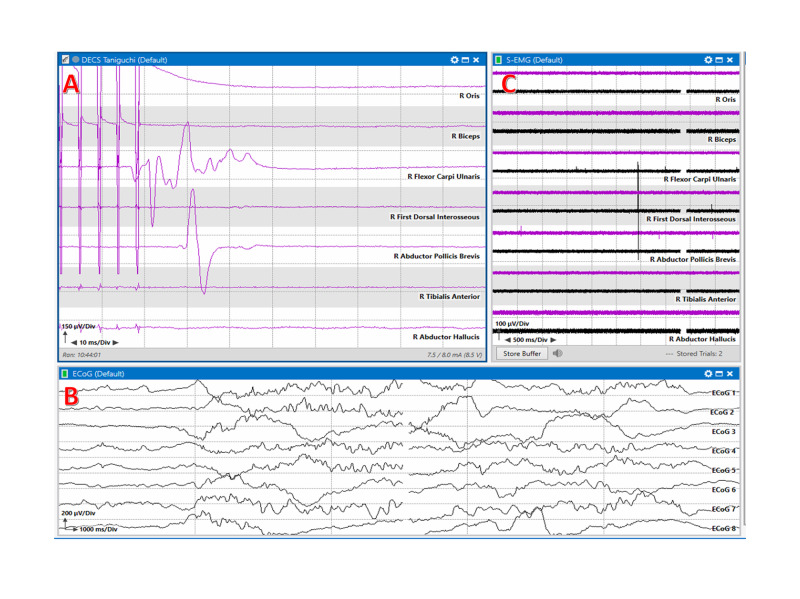
Multimodality mapping. Multimodality motor mapping with responses after monopolar handheld stimulation using a Taniguchi high-frequency method. A: Motor evoked responses are present in the right Flexor Carpi Ulnaris, and Abductor Pollicis Brevis muscles. B: Electrocorticography (ECoG), and C: Electromyography (EMG).

Electrocorticography (ECoG) is recorded intraoperatively by placing subdural grid electrodes and strips directly on the brain surface under the dura (Table 6). The spatial and temporal resolution of ECoG is higher than scalp EEG with no attenuation of the signal by the scalp and the skull. Therefore, the signal-to-noise ratio of ECoG is significantly better than scalp EEG. As compared to the scalp EEG, the ECoG waveforms are typically higher amplitude, higher frequencies, and can see dipole sources of both interictal and ictal activity. The subdural grids and strips electrodes are placed temporarily during the surgery to localize any epileptiform discharges (or after-discharges) during direct cortical stimulation (Figure 7). ECoG is also used to map and resect any epileptogenic regions of the brain (Figure 8). ECoG should be performed along with DECS for active tracking of after-discharges and preventing any seizure by immediately applying ice saline solution (4˚C) to the exposed cortex.

**Table 6:** Electrocorticography. Electrocorticography (ECoG) recording parameters. Hz = Hertz, µV = microvolts, div = division, ms = milliseconds, kΩ = kiloohms.

ECoG Recording Parameters	
Parameter	Value
Low-cut filter	1 Hz
High-cut filter	70 Hz
Notch filter	Off
Dynamic Range (Input Gain)	20 µV/div
Sensitivity	100 µV/div
Sweep	500 ms/div
Electrode impedance	> 5 kΩ

Train of four (TOF)

Train of four (TOF) is an additional important modality for consideration during motor mapping procedures involving the application of muscle relaxants. TOF is used to assess the level of the neuromuscular junction blockage due to muscle relaxants. Four pulses of stimulation per train are needed to facilitate response in selected peripheral nerves. The stimulation parameters are intensity between 10 and 100 mA, with a frequency of 2 Hz, pulse-width of 200 µs, stimulation duration of 2 seconds, an interstimulus interval of 0.5 ms, and an inter train interval of 10 seconds [12]. The recording parameter includes a sweep of 20 ms/div, with a gain of 100-500 µV/div (Table 7).

**Table 7:** Train of four. Train of four (TOF) responses representing the level of neuromuscular blockade during surgical anesthesia.

TOF Responses Present of Four Twitches	Degree of Neuromuscular Blockage
4 out of 4 responses	0 – 5%
3 out of 4 responses	65 – 75%
2 out of 4 responses	85%
1 out of 4 responses	95%
0 out of 4 responses	100%

Postoperative evaluation of patient

The patient should undergo evaluation based on the Medical Research Council (MRC) Scale score (M1 to M5), National Institutes of Health Stroke Scale score, and Karnofsky Performance Scale score. The patient needs to be evaluated for postoperative deficits immediately following surgery. We recommend that postoperative evaluations should be performed after 24 hours, 48 hours, two weeks, three months, and six months after the procedure.

## Discussion

The experience of the technologist and neurophysiologist is an essential factor for accurate motor mapping of the brain. Higher knowledge, experience, and comprehensive experience of the neurophysiological monitoring teams improve their ability to detect and preempt complications that might occur during the surgery [13].

According to Krieg et al., many cases of false negatives (4.5%) arise from ischemic or hemorrhaging events that occur postoperatively [14]. They are not false negatives from monitoring but instead are the result of accumulated damage to blood vessels during surgery that manifests after the neuromonitoring has concluded. Adequate training, proper equipment, and instruments are vital towards mitigating the risk of mistakes [6]. False positives are also critical as they impede surgical progress and can foster distrust in the surgeon towards future alerts. Potentially, it leads to loss of MEPs where proper care could have been taken to avoid such incidents [6]. Wrong interpretations also play their part in getting false results. It is highly recommended to use the predefined and well-established criteria provided by research and guidelines based on types of surgeries. Some of the alert criteria include the threshold of stimulation criteria, amplitude criteria, and morphology criteria [15-17]. Cedzich et al. corroborated the idea of employing EMG as a measure of intact motor pathways and mapping the cortex region, as it would not need the higher stimulation that inherently produces the risk of invoking seizure activity [18]. Also, the need to have to see limb movements to confirm the intact MEPs can be avoidable, especially for the microsurgical interventions. EMG allows for lower direct cortical stimulation to verify functional integrity.

Testing motor function via stimulating the motor strip under general anesthesia with the application of a bipolar stimulator was first employed by Fritsch and Hitzig. It is beneficial for motor mapping in cortical and sub-cortical regions, with better spatial stimulation resolution when compared to monopolar stimulation. However, it has been demonstrated that monopolar stimulation requires less amplitude for stimulation as it can directly activate pyramidal axons and induce repetitive excitation of the corticospinal tract (CST) while reducing the chance of damaging neural tissue [19].

## Conclusions

Intraoperative electrical stimulation of the corticospinal tract (CST) can be performed by two techniques, 50 Hz frequency Penfield and the high-frequency multipulse Taniguchi methods. Both methods provide a safe, helpful, and reliable resection near the central sulcus. A multimodality approach with sensory mapping, direct electrical cortical stimulation (DECS), electromyography (EMG), and electrocorticography (ECoG) increases the mapping accuracy at the lowest threshold with minimal risk of intraoperative seizures. Mapping of the motor cortex should be done after identifying the central sulcus with sensory mapping. DECS should be performed simultaneously with spontaneous EMG and ECoG to identify the ADs before the onset of seizures.

This review illustrates the technical details involved in intraoperative cortical motor mapping during brain surgeries. Following a set of standardized guidelines and taking steps with a clear and concise methodology inside the operating room helps patients come out of critical surgical interventions with minimal neurological deficits. The viability of employing advanced intraoperative neurophysiological monitoring techniques helps in guiding the surgeon with confidence and clarity. Involving the teams with experience in surgical procedures and IONM will have a better patient outcome as compared to less or non-experienced members. We propose that a multimodality approach towards neuromonitoring is necessary to minimize the probability of postoperative complications.
